# Supplementary material for: Crosstalk between Growth and Osmoregulation of GHRH-SST-GH-IGF Axis in Triploid Rainbow Trout (Oncorhynchus mykiss)
Source: Int J Mol Sci. 2022 Aug 4;23(15):8691. doi: 10.3390/ijms23158691 (PMC9369269; doi:10.3390/ijms23158691)
Supplement: Supplementary file 1 [file ijms-23-08691-s001.zip › ijms-1829016-supplementary.pdf]

Figure S1

A

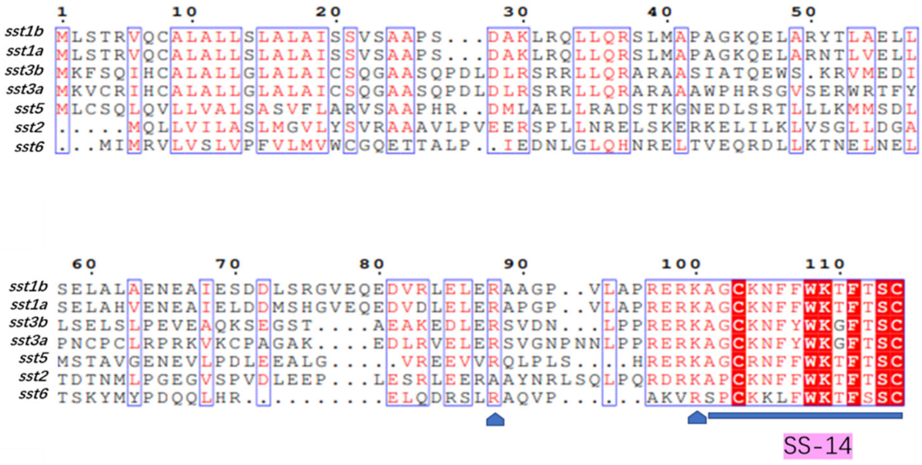

sst1b
sst1a
sst3b
sst3a
sst5
sst2
sst6
SS-14

B

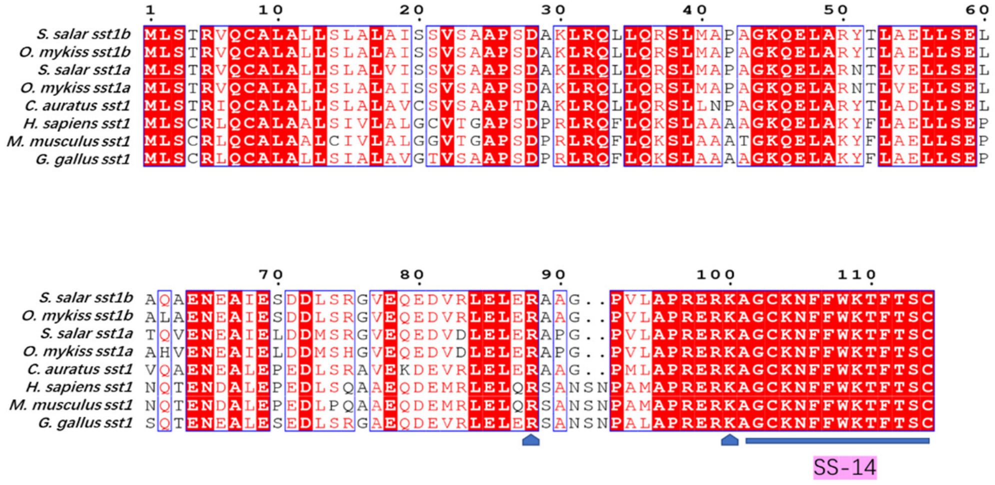

S. salar sst1b
O. mykiss sst1b
S. salar sst1a
O. mykiss sst1a
C. auratus sst1
H. sapiens sst1
M. musculus sst1
G. gallus sst1
SS-14

C

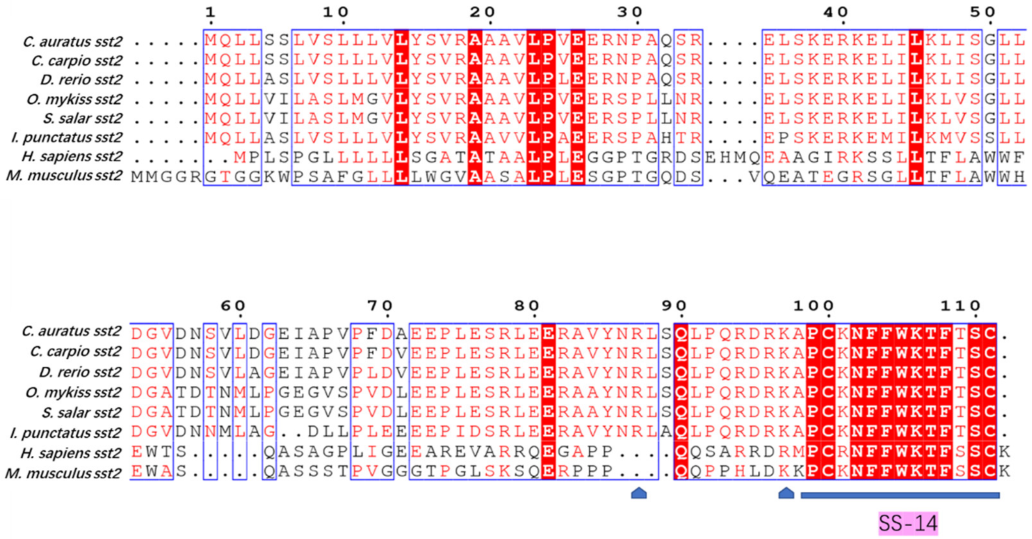

C. auratus sst2 .....MQLLSSLVSLLLVLYSVRAAAVLPVEERNPAQSR....ELSKERKELILKLISGLL
C. carpio sst2 .....MQLLSSLVSLLLVLYSVRAAAVLPVEERNPAQSR....ELSKERKELILKLISGLL
D. rerio sst2 .....MQLLASLVSLLLVLYSVRAAAVLPLEERNPAQSR....ELSKERKELILKLISGLL
O. mykiss sst2 .....MQLLVILASLMGVLYSVRAAAVLPVEERSPLLNR....ELSKERKELILKLVSGLL
S. salar sst2 .....MQLLVILASLMGVLYSVRAAAVLPVEERSPLLNR....ELSKERKELILKLVSGLL
I. punctatus sst2 .....MQLLASLVSLLLVLYSVRAAVVLPAEERSPAHTR....EPSKERKEMILKMVSSLL
H. sapiens sst2 .......MPLSPGLLLLLSGATATAALPLEGGPTGRDSEHMQEAAGIRKSSLLTFLAWWF
M. musculus sst2 MMGGRGTGGKWPSAFGLLLLWGVAASALPLESGPTGQDS...VQEATEGRSGLLTFLAWWH
C. auratus sst2 DGVDNSVLDGEIAPVPFDAEEPLESRLEERAVYNRLSQLPQRDRKAPCKNFFWKTFTSC.
C. carpio sst2 DGVDNSVLDGEIAPVPFDVEEPLESRLEERAVYNRLSQLPQRDRKAPCKNFFWKTFTSC.
D. rerio sst2 DGVDNSVLAGEIAPVPLDVEEPLESRLEERAVYNRLSQLPQRDRKAPCKNFFWKTFTSC.
O. mykiss sst2 DGATDTNMLPGEGVSPVDLEEPLESRLEERAAYNRLSQLPQRDRKAPCKNFFWKTFTSC.
S. salar sst2 DGATDTNMLPGEGVSPVDLEEPLESRLEERAAYNRLSQLPQRDRKAPCKNFFWKTFTSC.
I. punctatus sst2 DGVDNNMLAG..DLLPLEEEEPIDSRLEERAVYNRLAQLPQRDRKAPCKNFFWKTFTSC.
H. sapiens sst2 EWTS.....QASAGPLIGEEAREVARRQEGAPP....QQSARRDRMPCRNFFWKTFSSCK
M. musculus sst2 EWAS.....QASSSTPVGGGTPGLSKSQERPPP....QQPPHLDKKPCKNFFWKTFSSCK
SS-14

D

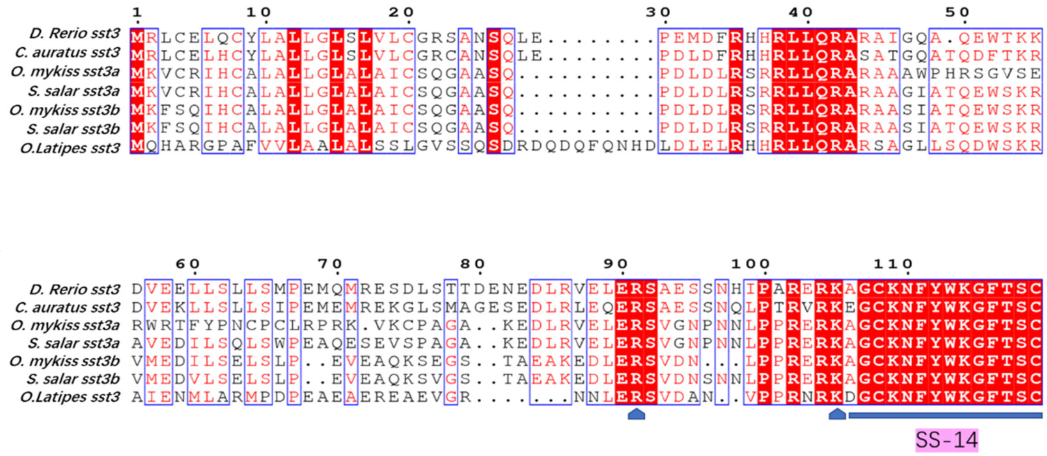

D. Rerio sst3 MRLCELQCYLALLGLSLVLCGRSANSQLE........PEMDFRHHRLLQRARAIGQA.QEWTKK
C. auratus sst3 MRLCELHCYLALLGLSLVLCGRCANSQLE........PDLDFRHHRLLQRASATGQATQDFTKR
O. mykiss sst3a MKVCRIHCALALLGLALAICSQGAASQ..........PDLDLRSRRLLQRARAAAWPHRSGVSE
S. salar sst3a MKVCRIHCALALLGLALAICSQGAASQ..........PDLDLRSRRLLQRARAAGIATQEWSKR
O. mykiss sst3b MKFSQIHCALALLGLALAICSQGAASQ..........PDLDLRSRRLLQRARAASIATQEWSKR
S. salar sst3b MKFSQIHCALALLGLALAICSQGAASQ..........PDLDLRSRRLLQRARAASIATQEWSKR
O.Latipes sst3 MQHARGPAFVVLAALALSSLGVSSQSDRDQDQFQNHDLDLELRHHRLLQRARSAGLLSQDWSKR
D. Rerio sst3 DVEELLSLLSMPEMQMRESDLSTTDENEDLRVELERSAESSNHIPARERKAGCKNFYWKGFTSC
C. auratus sst3 DVEKLLSLLSIPEMEMREKGLSMAGESEDLRLEQERSAESSNQLPTRVRKEGCKNFYWKGFTSC
O. mykiss sst3a RWRTFYPNCPCLRPRK.VKCPAGA..KEDLRVELERSVGNPNNLPPRERKAGCKNFYWKGFTSC
S. salar sst3a AVEDILSQLSWPEAQESEVSPAGA..KEDLRVELERSVGNPNNLPPRERKAGCKNFYWKGFTSC
O. mykiss sst3b VMEDILSELSLP..EVEAQKSEGS..TAEAKEDLERSVDN...LPPRERKAGCKNFYWKGFTSC
S. salar sst3b VMEDVLSELSLP..EVEAQKSVGS..TAEAKEDLERSVDNSNNLPPRERKAGCKNFYWKGFTSC
O.Latipes sst3 AIENMLARMPDPEAEAEREAEVGR........NNLERSVDAN..VPPRNRKDGCKNFYWKGFTSC
SS-14

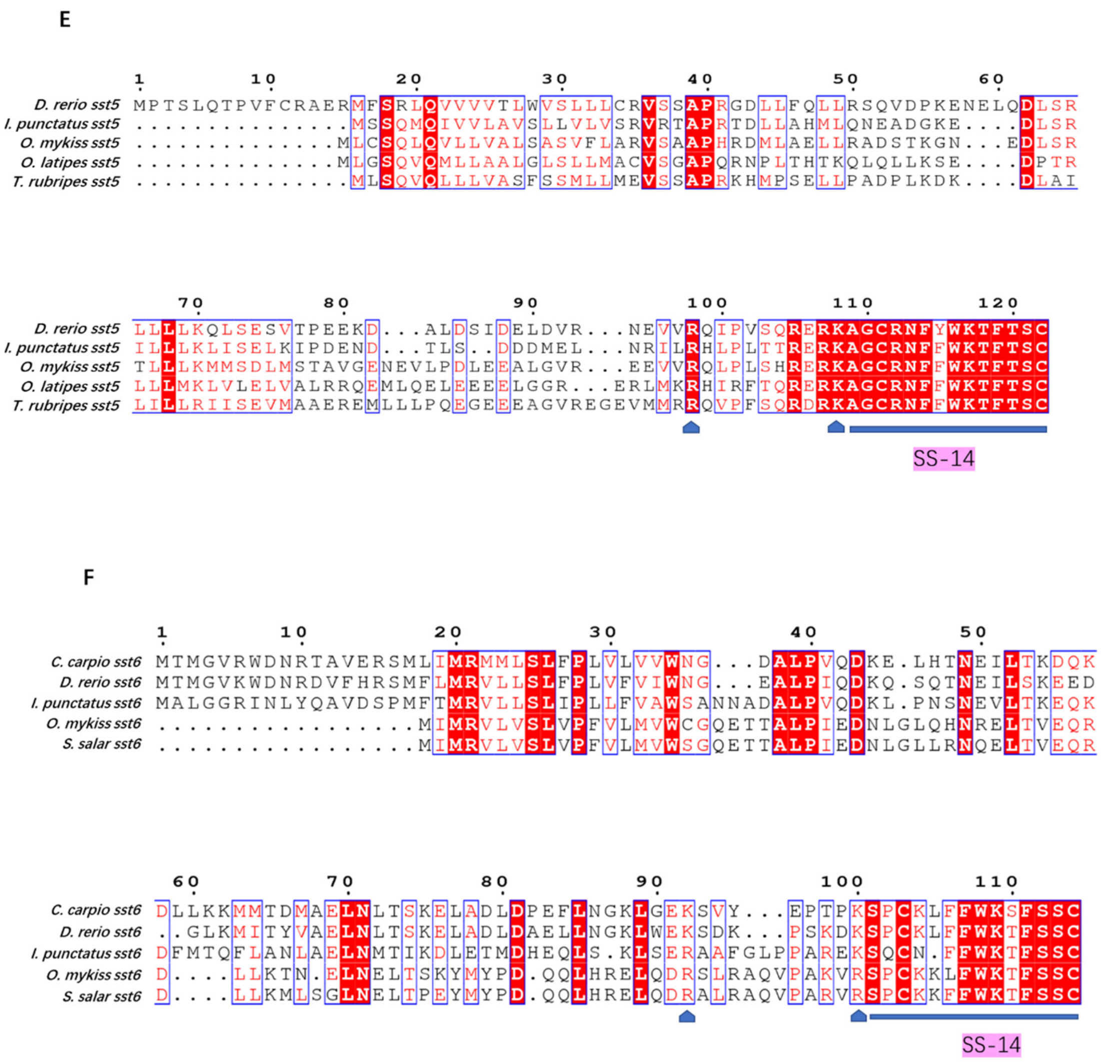

Figure S1. Sequence alignment of SST family in mammals and teleost. A: The SST amino acids in rainbow trout. B - F: SST amino acids in other vertebrates. The trout SST potential cleavage site is labeled with a purple arrow. The SS-14 peptide of the trout is underlined. The red represents the conserved amino acids.

Table S1. Expression matrix of *ghrh-gh-sst-igf* axis of brain and kidney.

Triploid				Diploid			
Gene	C_Brain1	C_Brain2	C_Brain3	Gene	C_Brain1	C_Brain2	C_Brain3
ghrh	1.938311	0	3.782739	*ghrh*	0.913913	1.025876	0
ghrhrl1	48.45776	56.77569	33.09897	*ghrhrl1*	31.07303	18.46577	26.89258
ghrhrl2	11.62986	13.18007	17.02233	*ghrhrl2*	17.36434	8.207008	26.89258
ghrhr2	2.907466	2.027703	0	*ghrhr2*	0	2.051752	0
gh1	0	0	0	*gh1*	0	0	0
gh2	0	0	0	*gh2*	0	0	0
ghra1	56.211	32.44325	78.49183	*ghra1*	74.02693	43.08679	54.90568
ghra2	17.44479	7.096961	7.565478	*ghra2*	25.58956	10.25876	6.723145
ghrb1	335.3277	388.3052	342.3379	*ghrb1*	140.7426	308.7887	309.2647

ghrb2	80.43989	58.80339	49.17561	*ghrb2*	64.8878	67.70782	36.9773
sst1a	156.034	63.87265	519.1809	*sst1a*	117.8947	29.75041	58.26725
sst1b	143.435	65.90035	226.9643	*sst1b*	281.4851	7.181132	4.482096
sst2	834.4427	598.1724	720.6118	*sst2*	1286.789	699.6475	742.9075
sst3a	0	0	0	*sst3a*	0	0	0
sst3b	50.39607	4.055406	52.01266	*sst3b*	17.36434	2.051752	0
sst5	324.667	335.5849	309.2389	*sst5*	79.51041	70.78545	86.28036
sst6	6.784087	0	4.728424	*sst6*	0	0	0
sstr1a1	61.05678	46.63717	71.87204	*sstr1a1*	43.86781	68.73369	106.4498
sstr1a2	127.9285	166.2717	156.038	*sstr1a2*	62.14606	174.3989	228.5869
sstr1b1	76.56327	97.32975	77.54615	*sstr1b1*	119.7226	151.8297	93.0035
sstr1b2	27.13635	23.31859	23.64212	*sstr1b2*	27.41738	11.28464	23.53101
sstr2a1	33.92043	9.124664	50.12129	*sstr2a1*	22.84782	16.41402	26.89258
sstr2a2	60.08763	48.66488	200.4852	*sstr2a2*	90.47736	32.82803	41.45939
sstr2b1	2.907466	4.055406	2.837054	*sstr2b1*	10.05304	2.051752	4.482096
sstr2b2	36.8279	29.4017	46.33855	*sstr2b2*	46.60955	18.46577	40.33887
sstr3a	0	1.013852	7.565478	*sstr3a*	8.225214	2.051752	3.361572
sstr3b	205.4609	51.70643	193.8654	*sstr3b*	57.5765	27.69865	23.53101
sstr5a	1.938311	0	1.891369	*sstr5a*	3.655651	6.155256	3.361572
sstr5b	1.938311	2.027703	5.674108	*sstr5b*	0.913913	0	4.482096
igf1a1	58.14932	79.08042	83.22026	*igf1a1*	52.09302	66.68194	48.18254
igf1a2	68.81002	43.59562	51.06698	*igf1a2*	59.40433	44.11267	43.70044
igf1b/igf3	13.56817	13.18007	24.5878	*igf1b/igf3*	10.05304	18.46577	8.964193
igf2	18.41395	19.26318	11.34822	*igf2*	62.14606	30.77628	19.04891
igf1ra1	134.7126	125.7176	91.73142	*igf1ra1*	142.5704	134.3898	134.4629
igf1ra2	120.1753	89.21894	71.87204	*igf1ra2*	179.1269	160.0367	137.8245
igfbp1a1	46.51945	38.52636	26.47917	*igfbp1a1*	46.60955	28.72453	48.18254
igfbp1a2	4.845776	5.069258	3.782739	*igfbp1a2*	17.36434	3.077628	6.723145
igfbp1b1	4.845776	1.013852	2.837054	*igfbp1b1*	0	0	0
igfbp1b2	1.938311	0	1.891369	*igfbp1b2*	5.483476	1.025876	0
igfbp2a1	189.9544	172.3548	151.3096	*igfbp2a1*	430.4529	242.1067	105.3293
igfbp2a2	168.633	117.6068	198.5938	*igfbp2a2*	241.273	179.5283	119.8961
igfbp2b1	0	1.013852	0	*igfbp2b1*	0	0	0
igfbp2b2	0	0	0	*igfbp2b2*	0	0	0
igfbp3a1	37.79705	43.59562	51.06698	*igfbp3a1*	95.04692	44.11267	62.74935
igfbp3a2	10.66071	2.027703	2.837054	*igfbp3a2*	3.655651	1.025876	1.120524
igfbp3b1	2.907466	1.013852	1.891369	*igfbp3b1*	6.397389	4.103504	1.120524
igfbp3b2	1.938311	8.110813	6.619793	*igfbp3b2*	28.33129	9.232884	8.964193
igfbp4a	625.1051	818.1782	953.2502	*igfbp4a*	1302.326	597.0599	646.5424
igfbp4b	380.878	522.1336	574.0306	*igfbp4b*	775.9119	485.2394	438.1249
igfbp5a1	46.51945	33.4571	68.0893	*igfbp5a1*	63.05998	23.59515	17.92839
igfbp5a2	48.45776	30.41555	166.4405	*igfbp5a2*	31.07303	12.31051	38.09782
igfbp5b1	130.836	118.6206	104.0253	*igfbp5b1*	279.6573	147.7262	105.3293
igfbp5b2	85.28566	76.03887	79.43752	*igfbp5b2*	171.8156	94.3806	67.23145
igfbp6a1	0.969155	3.041555	1.891369	*igfbp6a1*	0.913913	6.155256	6.723145
igfbp6a2	0.969155	1.013852	1.891369	*igfbp6a2*	0	0	0
igfbp6b1	77.53242	105.4406	93.62279	*igfbp6b1*	100.5304	68.73369	168.0786
igfbp6b2	42.64283	25.34629	49.17561	*igfbp6b2*	63.97389	23.59515	29.13363
actb	7516.768	10943.51	7687.471	*actb*	9072.411	17982.58	6792.617
Gene	C_Kidney1	C_Kidney2	C_Kidney3	Gene	C_Kidney1	C_Kidney2	C_Kidney3

ghrh	0	5.479102	0
ghrhrl1	143.3593	111.7737	144.0344
ghrhrl2	0	0	0
ghrhr2	0	0	0
gh1	0	0	0
gh2	0	0	0
ghra1	29.23776	12.05403	41.44874
ghra2	49.04398	29.58715	74.60774
ghrb1	416.8738	385.7288	539.8699
ghrb2	172.5971	156.7023	207.2437
sst1a	0	0	0
sst1b	10.37469	2.191641	5.181093
sst2	8.488381	17.53313	32.12277
sst3a	0	0	0
sst3b	0	0	0
sst5	8.488381	4.383282	4.144874
sst6	0	0	0
sstr1a1	9.431535	12.05403	10.36219
sstr1a2	0	0	0
sstr1b1	0.943153	3.287461	7.25353
sstr1b2	0	0	0
sstr2a1	0	0	0
sstr2a2	4.715767	7.670743	5.181093
sstr2b1	0	0	0
sstr2b2	5.658921	3.287461	7.25353
sstr3a	3.772614	3.287461	0
sstr3b	3.772614	6.574923	0
sstr5a	1.886307	2.191641	3.108656
sstr5b	2.82946	1.09582	5.181093
igf1a1	486.6672	139.1692	209.3161
igf1a2	160.3361	115.0612	115.0203
igf1b/igf3	95.2585	97.52802	126.4187
igf2	38.66929	26.29969	12.43462
igf1ra1	145.2456	93.14474	59.06446
igf1ra2	109.4058	35.06626	41.44874
igfbp1a1	26.4083	25.20387	14.50706
igfbp1a2	130.1552	127.1152	226.9319
igfbp1b1	0	0	0
igfbp1b2	35.83983	20.82059	11.3984
igfbp2a1	46.21452	85.474	59.06446
igfbp2a2	135.8141	316.6921	182.3745
igfbp2b1	0	3.287461	2.072437
igfbp2b2	0	0	0
igfbp3a1	17.91992	33.97044	16.5795
igfbp3a2	0	0	0
igfbp3b1	0	0	0
igfbp3b2	0	0	3.108656
igfbp4a	132.0415	164.3731	138.8533
igfbp4b	109.4058	106.2946	65.28177
igfbp5a1	7.545228	0	5.181093

ghrh	0	0.88769	0
ghrhrl1	43.43921	51.48603	69.80555
ghrhrl2	0.924238	2.663071	2.11532
ghrhr2	0	0	0
gh1	0	0	0
gh2	0	0	0
ghra1	27.72715	9.764593	17.98022
ghra2	90.57537	39.94606	37.0181
ghrb1	260.6352	346.1992	441.0442
ghrb2	181.1507	137.592	173.4562
sst1a	0	0	0
sst1b	2.772715	0.88769	2.11532
sst2	12.93934	34.61992	68.74789
sst3a	0	0	1.05766
sst3b	0	0	0
sst5	5.545431	0.88769	2.11532
sst6	0	0	4.23064
sstr1a1	9.242385	15.09073	3.17298
sstr1a2	0	0	0
sstr1b1	14.78782	1.77538	8.461279
sstr1b2	0	0	0
sstr2a1	0	0	0
sstr2a2	9.242385	4.438451	10.5766
sstr2b1	0	0	0
sstr2b2	1.848477	6.213832	14.80724
sstr3a	12.0151	0	2.11532
sstr3b	7.393908	0.88769	0
sstr5a	0.924238	7.989212	3.17298
sstr5b	0	0	2.11532
igf1a1	95.19656	82.55519	56.05597
igf1a2	18.48477	38.17068	19.03788
igf1b/igf3	107.2117	97.64593	97.30471
igf2	51.75735	63.9137	25.38384
igf1ra1	65.62093	57.69987	61.34427
igf1ra2	140.4842	71.01522	99.42003
igfbp1a1	23.10596	42.60913	66.63257
igfbp1a2	98.89352	18.6415	123.7462
igfbp1b1	0	0	0
igfbp1b2	40.66649	62.13832	70.86321
igfbp2a1	143.257	58.58756	98.36237
igfbp2a2	829.9661	412.776	654.6915
igfbp2b1	9.242385	0	3.17298
igfbp2b2	0	0	0
igfbp3a1	43.43921	32.84454	30.67214
igfbp3a2	2.772715	0	1.05766
igfbp3b1	0	2.663071	0
igfbp3b2	0	3.550761	0
igfbp4a	133.0903	99.42131	85.67045
igfbp4b	85.02994	124.2766	104.7083
igfbp5a1	12.0151	7.101522	11.63426

igfbp5a2	16.03361	17.53313	17.61572	*igfbp5a2*	9.242385	14.20304	22.21086
igfbp5b1	168.8245	111.7737	96.36832	*igfbp5b1*	218.1203	213.9333	127.9768
igfbp5b2	1820.286	1091.437	892.1842	*igfbp5b2*	1225.54	2164.189	761.5151
igfbp6a1	1.886307	0	2.072437	*igfbp6a1*	6.469669	3.550761	6.345959
igfbp6a2	33.01037	30.68297	35.23143	*igfbp6a2*	70.24212	65.68908	104.7083
igfbp6b1	315.0133	254.2304	240.4027	*igfbp6b1*	264.3322	200.618	192.4941
igfbp6b2	28.2946	16.43731	18.65193	*igfbp6b2*	34.19682	46.15989	31.7298
actb	10025.72137	30723.51898	12058.47519	*actb*	24617.09155	34981.20952	18861.24866

Table S2. Expression matrix of *ghrh-gh-sst-igf* axis of liver.

**Triploid**				**Diploid**						
Gene	TC_Liver1	TC_Liver2	TC_Liver3	Gene	CS1	CS2	CS3	CR1	CR2	CR3
ghrh	0.00	0.00	0.00	*ghrh*	0.00	0.94	1.26	0.98	0.00	0.00
ghrhrl1	112.83	73.71	65.21	*ghrhrl1*	73.91	80.79	88.45	49.77	48.44	39.21
ghrhrl2	0.00	0.00	0.00	*ghrhrl2*	0.00	0.00	0.00	0.00	0.00	0.00
ghrhr2	0.00	0.00	0.00	*ghrhr2*	0.00	0.94	0.00	0.00	0.00	0.00
gh1	0.00	0.00	0.00	*gh1*	0.00	0.00	0.00	0.00	0.00	0.00
gh2	0.00	0.00	0.00	*gh2*	0.00	0.00	0.00	0.00	0.00	0.00
ghra1	823.68	897.66	769.52	*ghra1*	706.45	892.49	622.94	741.67	346.13	540.62
ghra2	70.52	49.94	104.34	*ghra2*	161.94	126.83	69.50	71.24	27.25	67.06
ghrb1	412.78	359.06	567.36	*ghrb1*	762.96	551.47	624.20	1556.54	3212.06	1287.58
ghrb2	761.62	715.75	516.27	*ghrb2*	728.19	636.02	663.37	558.21	1042.43	594.27
sst1a	0.00	0.00	33.69	*sst1a*	42.39	0.00	0.00	119.06	0.00	0.00
sst1b	0.00	0.00	128.25	*sst1b*	110.86	6.58	1.26	30.25	2.02	1.03
sst2	0.00	0.00	0.00	*sst2*	0.00	0.00	0.00	0.00	0.00	0.00
sst3a	0.00	0.00	19.56	*sst3a*	39.13	4.70	0.00	7.81	0.00	0.00
sst3b	0.00	0.00	113.04	*sst3b*	42.39	0.00	0.00	34.16	0.00	3.10
sst5	2.82	4.76	0.00	*sst5*	8.69	1.88	2.53	0.00	11.10	0.00
sst6	0.00	0.00	0.00	*sst6*	0.00	0.00	0.00	0.00	0.00	0.00
sstr1a1	3.76	5.94	3.26	*sstr1a1*	4.35	2.82	1.26	0.98	2.02	0.00
sstr1a2	0.00	0.00	0.00	*sstr1a2*	0.00	0.00	0.00	0.00	0.00	0.00
sstr1b1	0.00	1.19	0.00	*sstr1b1*	2.17	0.00	0.00	0.00	0.00	1.03
sstr1b2	0.00	0.00	0.00	*sstr1b2*	0.00	0.00	0.00	0.00	0.00	0.00
sstr2a1	4.70	1.19	4.35	*sstr2a1*	1.09	19.73	17.69	0.00	0.00	1.03
sstr2a2	29.15	15.46	18.48	*sstr2a2*	8.69	29.12	30.33	33.18	14.13	3.10
sstr2b1	176.77	115.33	192.38	*sstr2b1*	383.66	224.53	169.32	125.89	120.09	200.15
sstr2b2	621.52	454.18	658.66	*sstr2b2*	1030.33	1069.11	1619.89	372.79	1052.52	779.98
sstr3a	0.00	0.00	0.00	*sstr3a*	0.00	0.00	0.00	0.00	0.00	0.00
sstr3b	0.00	0.00	0.00	*sstr3b*	0.00	0.00	0.00	0.00	0.00	0.00
sstr5a	1.88	2.38	8.70	*sstr5a*	9.78	1.88	2.53	4.88	6.05	15.48
sstr5b	9.40	4.76	7.61	*sstr5b*	20.65	8.46	0.00	16.59	4.04	1.03
igf1a1	984.47	2438.54	844.52	*igf1a1*	592.33	568.38	1079.08	240.07	330.99	335.31
igf1a2	552.88	1432.68	334.76	*igf1a2*	390.18	272.45	442.25	113.20	48.44	42.30
igf1b/igf3	15.04	20.21	17.39	*igf1b/igf3*	16.30	18.79	17.69	9.76	7.06	8.25
igf2	323.45	241.36	135.86	*igf2*	286.93	249.90	195.85	322.04	176.60	300.23
igf1ra1	0.00	9.51	2.17	*igf1ra1*	3.26	14.09	1.26	2.93	4.04	9.29
igf1ra2	5.64	4.76	3.26	*igf1ra2*	7.61	15.03	3.79	10.73	0.00	14.44

igfbp1a1	1716.94	450.61	1354.27	*igfbp1a1*	873.82	2328.00	3656.75	1381.85	6905.47	2556.59
igfbp1a2	126.94	97.49	148.90	*igfbp1a2*	88.03	322.24	399.29	113.20	538.88	140.31
igfbp1b1	708.97	193.80	4119.33	*igfbp1b1*	2825.79	1023.08	4293.58	3410.72	7732.96	1123.54
igfbp1b2	1716.94	789.46	3495.45	*igfbp1b2*	4603.87	3166.00	5881.88	1690.23	10322.38	2113.98
igfbp2a1	2320.60	2585.96	1919.46	*igfbp2a1*	6111.32	3435.63	4376.98	2677.83	1595.43	2421.43
igfbp2a2	3013.58	3465.79	2384.65	*igfbp2a2*	5260.32	4306.51	4871.03	2460.21	1344.16	1666.22
igfbp2b1	2058.26	1674.04	1556.43	*igfbp2b1*	1721.56	2226.53	3579.67	1555.56	1553.05	1469.16
igfbp2b2	0.94	3.57	3.26	*igfbp2b2*	16.30	0.00	3.79	0.00	0.00	0.00
igfpb3a1	0.00	0.00	1.09	*igfpb3a1*	0.00	2.82	0.00	0.00	0.00	0.00
igfpb3a2	0.00	1.19	0.00	*igfpb3a2*	5.43	3.76	0.00	1.95	0.00	3.10
igfbp3b1	0.00	0.00	2.17	*igfbp3b1*	6.52	2.82	0.00	0.00	0.00	0.00
igfbp3b2	0.00	0.00	0.00	*igfbp3b2*	0.00	0.00	0.00	0.00	0.00	0.00
igfbp4a	7.52	3.57	1.09	*igfbp4a*	8.69	4.70	12.64	7.81	4.04	19.60
igfbp4b	220.96	197.37	73.91	*igfbp4b*	52.17	125.89	102.35	13.66	0.00	7.22
igfbp5a1	9.40	10.70	3.26	*igfbp5a1*	32.61	36.64	59.39	9.76	65.59	27.86
igfbp5a2	9.40	33.29	16.30	*igfbp5a2*	19.56	36.64	15.16	12.69	20.18	5.16
igfbp5b1	67.70	51.12	53.26	*igfbp5b1*	223.89	118.37	123.83	59.53	49.45	126.90
igfbp5b2	8.46	2.38	14.13	*igfbp5b2*	17.39	20.67	5.05	0.98	1.01	2.06
igfbp6a1	0.00	0.00	0.00	*igfbp6a1*	0.00	0.00	0.00	0.00	1.01	0.00
igfbp6a2	6.58	9.51	10.87	*igfbp6a2*	6.52	33.82	11.37	18.54	7.06	12.38
igfbp6b1	409.96	332.91	310.85	*igfbp6b1*	420.61	334.45	452.35	500.63	901.15	827.44
igfbp6b2	10.34	10.70	6.52	*igfbp6b2*	39.13	31.00	7.58	5.86	0.00	1.03
actb	1532.65	2267.33	1049.94	*actb*	4485.40	2578.83	3276.41	549.42	1553.05	569.51

Figure S2

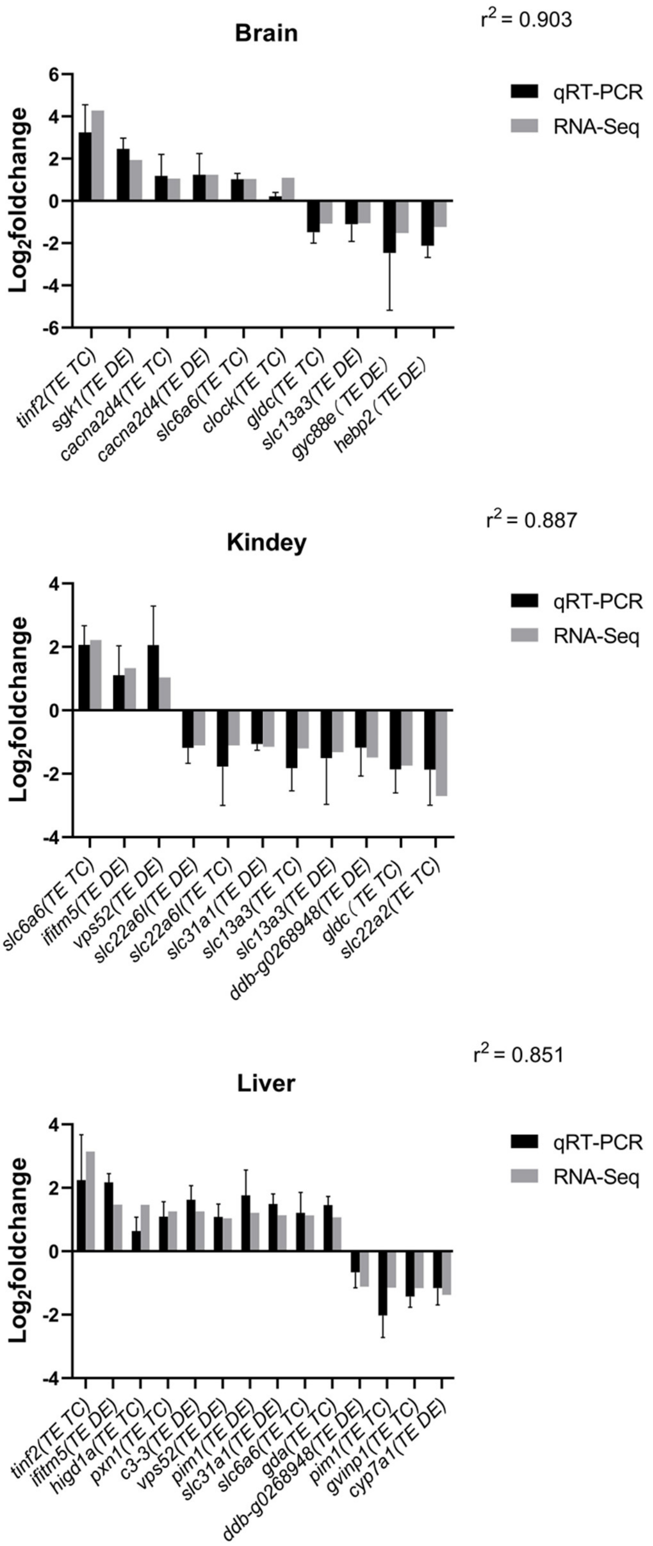

Figure S2. Validation of RNA-Seq results using qPCR. Apply Pearson correlation analysis in SPSS 22.0 to figure out the correlation coefficient r.

Table S3. Sequence of primers used for qPCR validation.

Gene	Forward (5′-3′)	Reverse (5′-3′)
tinf2	GCAGACGATGTCGAATGGC	CTACTGCTTGGGTCTCTGCG
slc6a6	CCACCGTTCTAAGGGCAAGG	ACAATAGTGTGGGTGGGCTT
gldc	CCATTCAAGAAGACCGCCA	TTCCCATCAACGCATCACA
sgk1	CTCTCAAGGACACATCATACTCAC	GCATCTCATAAAGCACGGC
slc13a3	CGCTACTTTTCGGGCTACCA	TCCCATGGTTGGAAAGAGG
hebp2	GAAGAGAAGAAGAAGATGTGGGC	CCATCCACCGTAGCTCCTCA
ifitm5	CGCCCAAGTTCCAACATAC	CACCGCTAACATTCCGAGA
c3-3	CCTCATACGCCCTGGCTAAC	TGTGGCCTCCAGAGTGAGA
cyp7a1	CAGTGGCCAAGAAAAGACGAC	CTCGTGCACTGCGAAGAAA
higd1a	CTGTGGGCTGAGATTCGTAG	ATGTCTCCACGGCTCTTCAA
ddb_g0268948	GCGAGTGCCAACTGGAGGA	AGCAAAGCCAAGCAGAGC[illegible]
cacna2d4	GGAGAGGGTAAAGTTGCGAA	GCCAGAGTGGAGATGTGTG
gyc88e	GAGAAACCCAAACGAGAGGAA	ACAGTCTGGAAGGCGAGCA
clock	GAGAAGAAGAGACGAGACCAGTT	TCATTATCGCTATGAAGAAT
vps52	TTGGACTTGACCACAGACGAG	GGTCAACACCCGTTTTCAGT
slc22a6l	TCTGTTGCAGAACTTTGTGGC	GTCTCGCTTCACCTTCGGAC
cldnk	GGATGACCTGCATCGTCCAA	AGAAAGCCGGAGATCCTTG
gda	TGTGGGCAAGGTGTGTATGG	GTTGGTTTCGTCATGGGACT
slc22a2	TCTGTTGCAGAACTTTGTGGC	GTCTCGCTTCACCTTCGGAC
gvinp1	TACCTTGGGGAGTTATTCG	TTGTTTGGCTTCCTTTGAG
pim1	TCTCCACCGTTGATATGCCG	GCCAGAATACACCGTCCCA
pxn1	GATAAAATGGGGCTGCTGGC	TTCTCATGGGCCTCTGGTTG
slc31a1	AGTACACAGTGCAGGAGGAATG	GATGACAAGACCGGCAAA[illegible]
